# Cross-cultural assessment of HIV-associated cognitive impairment using the Kaufman assessment battery for children: a systematic review

**DOI:** 10.7448/IAS.20.1.21412

**Published:** 2017-06-14

**Authors:** Kaylee S van Wyhe, Tanya van de Water, Michael J Boivin, Mark F Cotton, Kevin GF Thomas

**Affiliations:** ^a^ ACSENT Laboratory, Department of Psychology, University of Cape Town, Cape Town, South Africa; ^b^ Children with Infectious Diseases Clinical Research Unit, Department of Paediatrics and Child Health, University of Stellenbosch, Cape Town, South Africa; ^c^ Department of Psychiatry, University of Stellenbosch, Cape Town, South Africa; ^d^ Department of Psychiatry and Neurology and Ophthalmology, Michigan State University, East Lansing, MI, USA; ^e^ Department of Psychiatry, University of Michigan, Ann Arbor, MI, USA

**Keywords:** cognitive impairment, cross-cultural, HIV, Kaufman assessment battery for children, paediatric, systematic review

## Abstract

**Introduction**: Despite improved efficacy of, and access to, combination antiretroviral therapy (cART), HIV-associated cognitive impairments remain prevalent in both children and adults. Neuropsychological tests that detect such impairment can help clinicians formulate effective treatment plans. The Kaufman Assessment Battery for Children (KABC), although developed and standardized in the United States, is used frequently in many different countries and cultural contexts to assess paediatric performance across various cognitive domains. This systematic review investigated the cross-cultural utility of the original KABC, and its 2nd edition (KABC-II), in detecting HIV-associated cognitive impairment in children and adolescents.

**Methods**: We entered relevant keywords and MeSH terms into the PubMed, PsycInfo, EBSCOHost, ProQuest, and Scopus databases, with search limits set from 1983–2017. Two independent reviewers evaluated the retrieved abstracts and manuscripts. Studies eligible for inclusion in the review were those that (a) used the KABC/KABC-II to assess cognitive function in children/adolescents aged 2–18 years, (b) featured a definition of cognitive impairment (e.g. >2 SD below the mean) or compared the performance of HIV-infected and uninfected control groups, and (c) used a sample excluded from population on which the instruments were normed.

**Results and discussion**: We identified nine studies (eight conducted in African countries, and one in the United Kingdom) to comprise the review’s sample. All studies detected cognitive impairment in HIV-infected children, including those who were cART-naïve or who were cART treated and clinically stable. KABC/KABC-II subtests assessing simultaneous processing appeared most sensitive. Evaluation of the methodological quality of the selected studies by two independent reviews suggested that shortcomings included reporting and selection biases.

**Conclusions**: This systematic review provides evidence for the cross-cultural utility of the KABC/KABC-II, particularly the simultaneous processing subtests, in detecting cognitive impairment in HIV-infected children (including those who are clinically stable). Although the current results suggest there is justification for using the KABC/KABC-II primarily in East Africa, further investigation is required to explore the instrument’s utility in other HIV-prevalent regions of the globe.

## Introduction

Recent global estimates suggest that 3.2 million children under 15 years of age are living with HIV. Ninety-one per cent (more than 2.9 million) of those children reside in Sub-Saharan Africa [1]. The effects of HIV infection on children’s physical growth, psychological health, and neurodevelopment ranges from mild to devastating. These effects extend to cognitive development: A wealth of evidence indicates that HIV-infected children are likely to present with some form of cognitive impairment, with reported deficits in domains including attention, processing speed, language, motor skills, learning and memory, visual-spatial abilities, and executive functioning [2–4].

Research investigating the cognitive development of African, Indian, Asian, European, and South American HIV-infected children has reported a high (up to 90%) prevalence of cognitive and neurodevelopmental delays [5–11]. Despite this state of affairs, HIV-infected children are not routinely screened or formally assessed for cognitive delays or deficits. Although Boyede and colleagues [12] reported on the validation of a screening tool for rapid screening of moderate-to-severe global developmental delays in HIV-infected South African children, that tool is suitable only for those aged 9–36 months, and has not been validated for use elsewhere. Furthermore, although screening tools are useful in offering a basic determination of the presence or absence of cognitive deficits, they often lack the sensitivity and the theoretical framework required of comprehensive diagnostic instruments, and cannot deliver in-depth critical analysis of potential deficits [13,14].

Clinicians weighing the appropriateness of a cognitive measure for their particular context must consider whether a test developed and standardized on a specific population continues to measure the same construct when applied in a different setting [15,16]. Measured consideration of the cross-cultural equivalence of neuropsychological tests is often undermined by the grim practical reality of a severe lack of approved test material, however [17,18]. In low- and middle-income countries (LAMICs), especially, clinical neuropsychologists are hampered in their practice by a paucity of locally developed, standardized, and normed tests [19,20]. This situation is concerning in light of the prevalence of cultural and language differences, educational inequalities, and socio-political disadvantages that are often present in those countries, and that influence performance on standardized measures of cognitive function [21–23].

**Figure 1.**
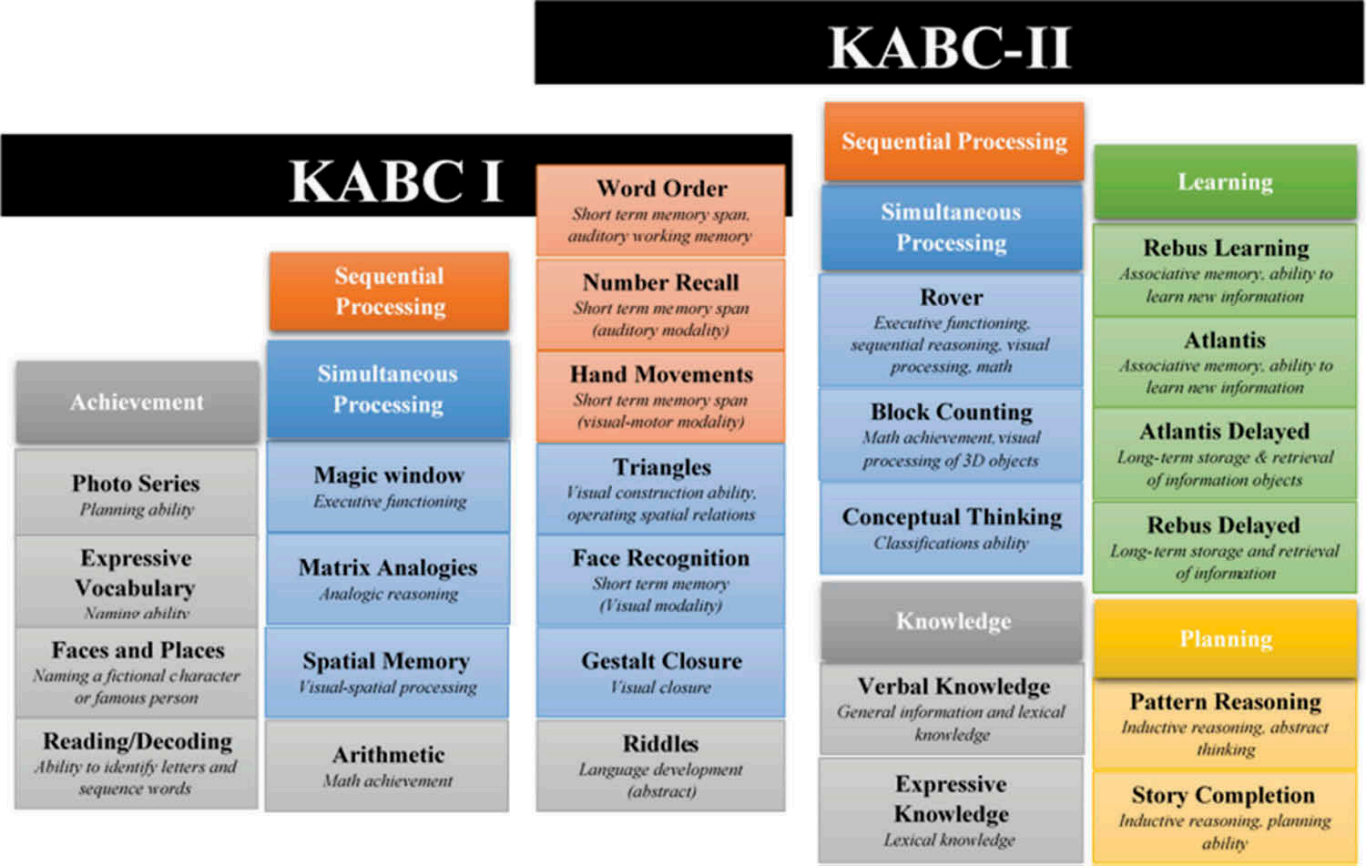
Subtests and indices comprising the KABC and KABC-II, and the cognitive abilities assessed by each. **The left panel shows subtests unique to the KABC, the right panel subtests unique to KABC-II, and the middle panel subtests common to the two. The KABC Achievement subtests (Photo Series, Expressive Vocabulary, Faces and Places, Reading/Decoding, Arithmetic, and Riddles), and the KABC-II Knowledge subtests (Verbal Knowledge, Expressive Knowledge, and Riddles) are shaded grey. These subtests assess crystallized knowledge. The KABC-II Learning subtests (Atlantis, Atlantis Delayed, Rebus, and Rebus Delayed) are shaded green. These subtests assess the ability to store and retrieve novel information. The KABC-II Planning subtests (Story Completion and Pattern Reasoning) are shaded yellow. These subtests assess the ability to solve nonverbal problems that require high-level decision-making and reasoning abilities. The KABC/KABC-II Sequential Processing subtests (Hand Movements, Number Recall, and Word Order) are shaded orange. These subtests assess the ability to solve problems by coding auditory and visual information presented serially. Simultaneous Processing subtests of the KABC (Magic Window, Matrix Analogies, Spatial Memory, Arithmetic, Triangles, Face Recognition, and Gestalt Closure) and of the KABC-II (Rover, Block Counting, Conceptual Thinking, Triangles, Face Recognition, and Gestalt Closure) are shaded blue. These subtests assess the ability to solve spatial or logistical problems that require the processing of many related stimuli simultaneously. Summing scores across these subtests/indices generates a Mental Processing Index (MPI) score, which reflects the child’s overall performance on the battery. On the KABC-II, summing scores across the Hand Movements, Block Counting, Triangles, Pattern Reasoning, Story Completion, Conceptual Thinking, and Face Recognition subtests generates a Nonverbal Index (NVI) score. This set of subtests is used in children for whom a nonverbal measure of cognitive functioning is deemed appropriate (e.g. those with severe speech or language deficits).**

The Kaufman Assessment Battery for Children (KABC), and its revised second edition, the KABC-II, are measures of intellectual functioning, developed and standardized in the United States, with strong psychometric characteristics [24,25]. As Figure 1 shows, the KABC and KABC-II both assess a wide range of cognitive domains, including those commonly affected by HIV in children. The KABC is suitable for administration to children aged from 2 years 6 months to 12 years 6 months, whereas the KABC-II is suitable for children aged from 3 years 0 months to 18 years 11 months. Each battery can be administered in 25–100 min, depending on the child’s age. Whereas the KABC was grounded in the Horn and Cattell’s theory of crystallized versus fluid intelligence [26], the KABC-II’s results can be interpreted according to either Luria’s neuropsychological theory of processing [27] or the Cattell-Horn-Carroll (CHC) psychometric model [28]. What this means, in practice, is that the KABC-II measures the same abilities as the KABC, but also measures abilities in two additional cognitive domains (viz., Planning and Learning).

Both the KABC and KABC-II have been used across the globe to assess cognitive functioning comprehensively [29,30]. Their widespread use is attributable largely to the fact that (a) they incorporate teaching items to increase familiarity with the test materials, (b) test responses require very little verbalization from examinees, and (c) early psychometric studies suggested they were culture-fair when applied to different ethnic groups within the United States [31–33]. Subsequently, validation studies conducted in Africa and Asia have demonstrated that the instruments maintain their construct validity, and are sensitive to socio-economic factors and disease effects [e.g. 34,35,36]. Furthermore, a meta-analysis of KABC validation studies across cultures supported the factor integrity of the distinction between the Sequential Processing versus Simultaneous Processing indices [37]. In summary, strong theoretical and psychometric foundations, culture-fair assessment techniques, and cross-cultural adaptability have positioned the KABC/KABC-II as the best-choice instrument for researchers or clinicians who operate in a variety of cultural contexts and who require a well-standardized measure of cognitive ability within specific domains [30,36,38].

Despite the KABC/KABC-II’s popularity, and its widespread use in regions where HIV is highly prevalent (e.g. sub-Saharan Africa), no study has formally evaluated whether these instruments are suited to identify cognitive impairment, across distinct and independent domains, in HIV-infected children. (Indeed, there are currently no validated neuropsychological tests, or test batteries, designed specifically to detect such deficits in children or in adolescents.) Hence, this systematic review aimed to determine whether the KABC/KABC-II identifies HIV-associated cognitive impairment in children who reside in cultural contexts outside of that in which the instrument was developed, standardized, and validated.

## Methods

**Figure 2.**
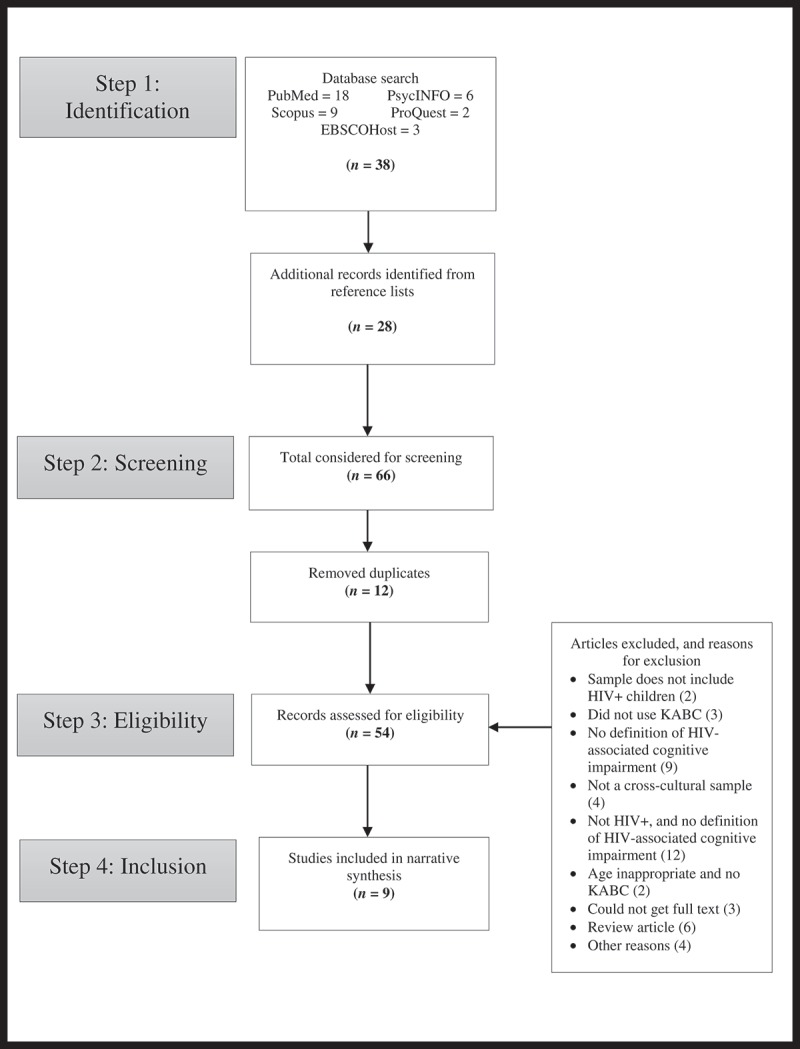
PRISMA flowchart documenting search process and results.

Figure 2 is a PRISMA flowchart documenting the process by which we arrived at the final sample of studies that met the eligibility criteria and that were included in the systematic review. Below, we describe each stage of that process in detail.

### Search strategy

We used electronic databases (EBSCOHost, ProQuest, PsycInfo, PubMed, and Scopus) to search for published and unpublished studies, posters, PowerPoints, and abstracts. The search limits were set from 1983 (the year the KABC was published) to February 2017. Keywords were MeSH and non-MeSH search terms covering HIV/AIDS, children, HIV medication (e.g. HAART), KABC, and cognitive development/functioning (see Additional File 1). The search identified 38 studies. We then conducted a manual search of the references from the identified articles and published conference proceedings to ensure all relevant articles were identified. This arm of the search strategy yielded an additional 28 articles.

### Study selection

Two authors (KvW and TvdW) screened abstracts (and full text if needed) to determine inclusion status. Disagreements were resolved by discussion. We included studies that met the following criteria: (1) Used a sample that comprised HIV-infected children (who may or may not have been on cART, and may or may not have been co-infected); (2) used a sample consisting of children between the ages of 2 and 18 years, inclusive; (3) used (a subtest of) the KABC/KABC-II; (4) provided a method to indicate cognitive impairment in the HIV-infected sample (e.g. comparison to normative or control group, definition of impairment provided); and (5) used a sample excluded from the original normative data on which the KABC was standardized (i.e. we excluded studies that used as their samples individuals who identified as African-American, Hispanic, American White, American Indian, Alaskan Native, Asian-American, or Pacific Islander).

There were no exclusion criteria based on language or format of publication. All study designs were included, with the exception of reviews. The latter were used to search for any other studies that could be included. If a full text was unavailable online, we emailed the authors and followed up weekly. If there was no response after 3 months, the article was considered excluded/missing data. Items coded as “not specified” (NS) indicates that we did not receive a response (see Additional File 2).

### Data extraction and quality assessment

Nine studies (seven peer-reviewed journal articles, one poster, and one Master’s thesis) fulfilled the inclusion criteria and were reviewed [39–47]. Two authors (KvW and TvdW) performed data extraction using a spreadsheet (see Additional File 1) based on the STROBE statement for observational studies [48]. STROBE is a detailed checklist developed to ensure that adequate data are extracted from all studies (e.g. cohort, cross-sectional, case-control) included in systematic reviews. Here, we were particularly interested in the place of study, inclusion of confounding variables, reporting of study limitations and strengths, and detailed information on HIV status and KABC results. In addition, the same two authors independently assessed the quality of all included studies (see Table 1 and Additional File 1) using a modified version of the Downs and Black [49] checklist. Originally designed for the assessment of the methodological quality of randomized and non-randomized studies, the checklist consists of 26 items representing six sub-scales: reporting, internal validity, external validity, bias, confounding, and power. Because none of the included studies reported power calculations to determine whether there was an adequate sample size to assess the ability of the KABC to determine between-group differences, item 27 was excluded from the standard checklist. Hence, the maximum score for the modified checklist was 26. Again, disagreements were resolved by discussion. Since the Downs and Black checklist does not stipulate a cutoff for suitable studies, we used the mid-point score of 13 to differentiate between lower- and higher-quality studies [50].

**Table 1.** Study Quality Assessment

Study ID	Study Site	Study Type	Quality^a^	Confounding Variables	Primary Limitations	Primary Strengths
Bagenda et al. [39]	Uganda	Cross-sectional	22	Age, sex, HAZ, WAZ, cranial nerve function	Potential selection bias noted.	None of subjects’ mothers had received ARVT or been exposed to illicit drugs. Testers were blinded.
Boivin et al. [40]	Uganda	Cross-sectional	21	Age, sex, weight, CD4, viral load, HOME score	Does not describe participant selection process.	Testers were blinded. KABC-II validated in/for Uganda. Used local normative data to compare results.
Boivin et al. [41]	Congo	Cross-sectional	15	Age, height, weight, head circumference, arm circumference, Quaker arm circumference	Does not report exact *p* values for the main outcomes, except where values are <.05/.01/.0001. Unable to determine if subjects were representative of the population from which they were recruited. Unable to determine attempts were made to blind those assessing participants.	Local HEU and HUU control groups used for statistical comparison of results.
Boivin et al. [47]	Multi-site^b^	Prospective	13	Age, sex, race, height, weight, BMI, caregiver educational level, who caregiver is, sibling enrolled in study	Poster format, hence underreporting of required information (e.g. representativeness of sample, whether testers were blinded, recruitment procedures, test adaptation).	Multi-site study with large sample size.
Boivin et al. [42]	Uganda	RCT	21	Age, sex, WAZ, SES, pre-intervention Cogstate score	Unable to determine if attempts were made to blind those assessing subjects.	KABC-II validated for children in Ugandan context.
Brahmbhatt et al. [43]	Uganda	Longitudinal	16	Age, sex, HAZ, WAZ, grade at school	Participant loss to follow up not well described. Unable to determine if those conducting assessments were blinded.	KABC-II validated for children in Ugandan context.
Gosling et al. [44]	UK	Longitudinal	14	CD4, viral load	Statistical tests and results/probabilities are not reported. Unable to determine if loss to follow-up was taken into account statistically. Unable to determine if subjects are representative of the entire population from which they were recruited. Small sample size.	Interventions and principal confounders are clearly described.
Merkle [45]	South Africa	Longitudinal	22	Sex, hand preference, nutritional status, grade, home language, SES, caregiver health status/educational level	Does not state whether CD4 and viral loads were taken into account for statistical analysis.	Clear description of recruitment process.
Ruel et al. [46]	Uganda	Cross-sectional	17	Height, weight, WHO stage, CD4, plasma HIV RNA level, SES	Does not state whether the subjects asked to participate were representative of entire population from which they were recruited.	Distributions of principal confounders in each group of subjects described clearly. KABC-II validated for Ugandan children

ARVT: antiretroviral treatment; BMI: body mass index; HAZ: height-for-age *z*-scores; HEU: HIV-exposed uninfected; HOME: Caldwell Home Observation for the Measurement of the Environment; HUU: HIV-unexposed uninfected; RCT: randomized control trial; SES: socio-economic status; UK: United Kingdom; WAZ: weight-for-age *z*-scores; WHO: World Health Organization.

^a^Score on Downs and Black checklist, where the maximum possible score is 26.

^b^Includes South Africa (Cape Town, Johannesburg, Soweto), Malawi, Uganda, & Zimbabwe.

## Results and discussion

We set out to determine, via systematic review, whether a popular, widely used, and psychometrically sound cognitive test battery, the Kaufman Assessment Battery for Children, identifies HIV-associated cognitive impairment in children who reside in cultural contexts outside of that in which the instrument was developed, standardized, and validated.

### Study characteristics

As Table 1 shows, 8 of the 9 studies that formed the final sample were conducted in Africa, where the vast majority of HIV-infected children reside. The other study was conducted in the United Kingdom. Uganda was the most represented country (five studies), followed by South Africa (two studies, one of which was a multi-site study that also featured data collected from Malawi, Uganda, and Zimbabwe), England, and the Democratic Republic of the Congo (one each). Four studies featured cross-sectional designs, three were longitudinal, one used a prospective design, and one was a randomized control trial.

As Table 2 shows, the studies featured a total of 1792 participants, including 720 (51%) who were HIV infected. Of the latter participants, 329 (46%) were cART-naïve at study initiation. Across all studies, the age range of participants was 2–14 years (*M* for HIV-infected participants = 7.9 years; *M* for controls, across the seven studies that reported this statistic, was 7.4 years). Regarding the sex distribution across the comparison groups, in the six studies that reported this statistic 369 of the 720 HIV-infected participants (51%) were female, as were 473 of the 842 uninfected controls (56%).

**Table 2.** Description of study characteristics and findings

		*n*_HIV+_ (%cART-naïve);	*n*_HIV-_ (% HEU);	KABC				Domain
		*M* age (yrs);	*M* age (yrs);					
Study ID	*N*	*n*_female_ (%)	*n*_female_ (%)	Version	Test Adaptations	Test Administrator	Specific Results^a^	of Impairment
Bagenda et al. [39]	107	28 (100%);	79 53%);	I	• Language adapted	Child psychometrist	• Hand Movements (*p *= .02)	• Visual STM
		9.1;	8.7;		• Knowledge component not administered			
		18 (64%)	42 (53%)					
Boivin et al. [40]	176	54 (100%);	122	II	• Language adapted	Native speakers	• Seq. Processing	• Memory
		9.0	(NR);		• Knowledge component not administered		• Sim. Processing	• VS
		NR	NR;				• Learning	• IR/DR
			NR				• Planning	• EF
Boivin et al. [41]	41	11 (100%);	30 50%);	I	• Language adapted	Local teachers	• MPI (*p *< .0001)	• Global
		4.6;	2.0;		• Only Mental Processing subtests (except Photo Series) administered		• NVI (*p *< .05)	
		NR	NR				• Sim. processing (*p *< .0001)	
							• Seq. processing (*p *< .0001)	
Boivin et al. [47]	611	246 (0%);	365 (50%);	II	NR	Research assistants	• MPI • NVI (*p *< .0001)	• Global
		7.0;	6.8;					
		135 (55%)	186 (51%)					
Boivin et al. [42]	166	60 (95%);	106	II	NR	NR	• Seq. processing (*p *< .01)	• Memory
		9.9;	(NR);				• Sim. processing (*p *< .002)	• VS
		36 (60%)	8.8;				• Learning (*p *= .05)	• IR/DR
			66 (62%)					
Brahmbhatt et al. [43]	370	140 (9%);	230 1%);	II	• Knowledge component administered	Nurses and Midwives	• Sim. Processing (*p *= .035)	• VS
		8.6;	9.9;				• Learning (*p* = .047)	• IR/DR
		75 53%)	120 2%)				• Knowledge (*p* < .001)	• Language
							• NVI (*p* < .001)	
Gosling et al. [44]	11	11 36% at Time 1,	0	I	• Achievement scale	Psychologists	NR	NR
		18% at Time 2);			not administered			
		7.3;						
		3 (27%)						
Merkle [45]	111	77 0%);	34 50%);	II	• Language adapted	Research assistants	• MPI (*p *= .002)	• Global
		7;	7;		• Knowledge component not administered		• NVI (*p *= .025)	• `VS
		44 57%)	14 41%)				• Sim. Processing (*p *= .001)	• IR/DR
							• Learning (*p *< .001)	• EF
							• Planning (*p *= .007)	
Ruel et al. [46]	199	93 100%);	106 R);	II	• Language adapted	NR	• Seq. processing (*p *= .005)	• Memory
		8.7;	8.7;		• Knowledge component not administered		• Sim. processing (*p *= .039)	• VS
		58 (62%)	45 42%)				• Planning (*p *= .023)	• EF

cART: combination antiretroviral therapy; EF: executive functioning; HEU: HIV-exposed uninfected; IR/DR: immediate recall and delayed recall; MPI: Mental Processing Index; NR: not reported; NVI: Nonverbal Index; Seq.: sequential; Sim.: simultaneous; STM: short-term memory; VS: visual-spatial reasoning and problem solving.

^a^KABC I/II subtests/indices on which HIV-infected participants performed statistically significantly more poorly than controls. *p*-values are reported when presented in the original.

Four studies [39,41,43,44] used the KABC, whereas the rest used the KABC-II. All adapted the instrument’s administration and/or scoring to improve its fairness to their sample. For instance, six [39–41,44–46] did not administer (or, at least, do not report results related to) the Achievement and/or Knowledge subtests, which assess crystallized intelligence and therefore are likely to rely heavily on exposure to the mainstream culture within which the test was developed [51]. Similarly, five [39–41,45,46] reported administering the instrument in the participants’ home language.

### Quality assessment

Table 1 presents the findings from our critical evaluation of the quality of each of the eight studies. Most were rated as being of relatively higher methodological quality, with all except one scoring above the mid-point score of 13 on the Downs and Black checklist, and four scoring more than 20 out of the maximum possible 26. All studies considered potentially confounding variables (e.g. age, sex, CD4 and viral loads, and whether participants were cART treated or cART naïve) in their interpretation of results, and all except one included such potential confounders in their statistical analyses. During data extraction, we noted that reporting limitations primarily related to the selection and recruitment of samples. Hence, we cannot eliminate the possibility of selection bias based on the information provided in the articles. We also noted that, in two of the eight studies, some results were based on data dredging.

### KABC/KABC-II identification of HIV-associated cognitive impairment

Overall, our review suggests that the KABC/KABC-II can be used successfully across different countries and cultural contexts to identify cognitive impairment in HIV-infected children and adolescents. Hence, although there is no current consensus regarding whether adult diagnostic criteria for HIV-associated neurocognitive disorders (HAND) might be applied to children and adolescents [52], it appears the KABC/KABC-II might usefully serve, alongside assessments of functional competency, as a core component of a battery that describes where along the HAND spectrum HIV-infected individuals younger than 18 years might be placed.

In each of the reviewed studies, the KABC/KABC-II successfully identified cognitive impairment in HIV-infected children, either relative to uninfected counterparts or to their own baseline. Eight of the nine studies used local normative data, or a local reference group, against which to compare cognitive performance of HIV-infected children. These studies identified impairment at the group level (i.e. they classed the performance of the group of HIV-infected children as “impaired” if there were significant between-group differences, in favour of the control/normative group, on the particular subtest or index under consideration). Gosling et al. [44] reported a decline in cognitive functioning across longitudinal follow-up, but did not specify (a) whether this decline suggested impairment relative to healthy controls, or (b) the subtests/scales that formed the bases for this observation. As Table 2 shows, six studies reported significant between-group differences on the Simultaneous Processing index, suggesting that HIV-infected children might have particular difficulty on visual-perceptual tests that require them to disintegrate, manipulate, and reintegrate component parts of a whole unit. Together with the fact that four studies also detected significant between-group differences on the Sequential Processing index (i.e. on tests that assess the ability to encode, store, and then organize items of information into a logical sequence), one might conclude that the cognitive processes involving activity in the posterior regions of the brain are particularly susceptible to impairment in HIV-infected children [32,52].

Across all studies, there was no single KABC/KABC-II subtest or scale on which HIV-infected children performed consistently poorly. Hence, this review suggests that no single subtest offers the potential to be adapted into a stand-alone screening tool. This conclusion is consistent with research indicating that numerous independent cognitive domains are affected in HIV-infected children [3,53]. Appropriate assessment of these children should therefore include administration of, at least, Simultaneous Processing, and Sequential Processing subtests of the KABC/KABC-II, and should probably include the Planning and Learning subtests as well.

Our review also demonstrates that the KABC/KABC-II is sensitive to cognitive impairment in HIV-infected children, with and without cART, when compared to controls. This finding is promising because a growing body of research demonstrates that subtle cognitive impairments may persist even in HIV-infected children who are well controlled on cART [54–56].

To improve the culture-fairness of the instrument, researchers across the reviewed studies typically implemented a three-part strategy: (1) They translated it into the local language; (2) they excluded either or both the Knowledge and Achievement components (i.e. those subtests that rely heavily on crystalized intelligence, or learned, culture-specific environmental experiences); and (3) where local normative data were unavailable, they applied either conventional cutoffs (1 SD below the standardization sample mean), or stricter cutoffs (2 SD below the mean of a local control group), to classify impairment, depending on whether standardization sample data were judged applicable or not.

Finally, we identified an interesting trend in the reviewed studies: The KABC/KABC-II was not always administered by a registered or licensed clinical psychologist or neuropsychologist. Rather, administration fell to local teachers, research assistants, or psychometrists. Although not all of the manuscripts make it clear, perusal of the author lists and acknowledgements suggests that these test administrators all operated under appropriate supervision. In light of the scarcity of highly-trained professionals in low- and middle-income countries where HIV is prevalent [39,57], it is useful to know that the instrument can be administered by trained lay professionals, with the ethical proviso that these individuals work (a) according to guidelines offered by the International Test Commission, (b) under the supervision of a qualified expert, and (c) with permission of the test publisher.

### Limitations

The strength of the conclusions one might draw from this systematic review are limited by the characteristics of the reviewed studies and by the nature of the reviewed instrument. We therefore offer the following caveats.

First, although authors of the included studies noted that they had translated the instrument from English into the local language, most did not describe their translation procedures in detail. So, for example, it is unknown whether rigorous back-translations procedures were in place, and whether community members were consulted about idiomatic aspects of the translation. The absence of this information raises questions around linguistic equivalence of the various translated versions of the KABC/KABC-II.

Second, no KABC/KABC-II subtest measures the cognitive construct of information processing speed directly. This limits the value of the instrument in assessment of HIV-infected children in different countries and cultural contexts, given that (a) strong recent evidence suggests that processing speed is an important component of HANI [53], and (b) there are cross-cultural differences in the rate at which processing speed matures and develops across childhood and adolescence [58].

Third, HIV-1 subtype distribution is not consistent across the globe [7], and so the studies reviewed here were not focused on cognitive impairment associated with a single clade type. For instance, the studies in Central and East Africa likely included a predominance of clade A-infected children, whereas those conducted in sub-Saharan Africa likely included a predominance of clade C-infected children. Hence, one must exercise caution when generalizing these findings because of the possibility that clade-specific neuropathogenic differences might manifest in differing degrees of disease severity [40,59,60].

## Conclusions

The findings of this review suggest that the KABC/KABC-II has cross-cultural utility. It appears that the instrument can provide comprehensive information regarding cognitive impairment in HIV-infected children, regardless of the country or cultural context in which it is administered. The instrument is especially useful because it can be administered by laypersons, and because it is sensitive enough to identify impairment in children who are otherwise well managed (i.e. who are clinically stable on cART). However, the review also highlights the need for more cross-cultural validity studies of the KABC/KABC-II, and, particularly, for research investigating whether the instrument is sensitive to clade-specific variations in cognitive impairment. In such future research (and, indeed, in any research using the KABC/KABC-II with HIV-infected children), we suggest that the adaptation procedures described in the studies reviewed here be used as a baseline to ensure culture-fair testing. We further recommend that, when adapting test material, researchers apply the standard procedures set out by the International Test Commission [61,62], and that they describe all adaptations clearly in the published material.

## Notes

To access the supplementary material to this article please see Supplementary Files under Article Tools online.
